# Monitoring the Nutrient Composition of Food Prepared Out-of-Home in the United Kingdom: Database Development and Case Study

**DOI:** 10.2196/39033

**Published:** 2022-09-08

**Authors:** Yuru Huang, Thomas Burgoine, Michael Essman, Dolly R Z Theis, Tom R P Bishop, Jean Adams

**Affiliations:** 1 Medical Research Council (MRC) Epidemiology Unit University of Cambridge Cambridge United Kingdom

**Keywords:** nutritional database, web scraping, food prepared out of the home, out-of-home, data science, chains

## Abstract

**Background:**

Hand transcribing nutrient composition data from websites requires extensive human resources and is prone to error. As a result, there are limited nutrient composition data on food prepared out of the home in the United Kingdom. Such data are crucial for understanding and monitoring the out-of-home food environment, which aids policy making. Automated data collection from publicly available sources offers a potential low-resource solution to address this gap.

**Objective:**

In this paper, we describe the first UK longitudinal nutritional database of food prepared out of the home, MenuTracker. As large chains will be required to display calorie information on their UK menus from April 2022, we also aimed to identify which chains reported their nutritional information online in November 2021. In a case study to demonstrate the utility of MenuTracker, we estimated the proportions of menu items exceeding recommended energy and nutrient intake (eg, >600 kcal per meal).

**Methods:**

We have collated nutrient composition data of menu items sold by large chain restaurants quarterly since March 2021. Large chains were defined as those with 250 employees or more (those covered by the new calorie labeling policy) or belonging to the top 100 restaurants based on sales volume. We developed scripts in Python to automate the data collection process from business websites. Various techniques were used to harvest web data and extract data from nutritional tables in PDF format.

**Results:**

Automated Python programs reduced approximately 85% of manual work, totaling 500 hours saved for each wave of data collection. As of January 2022, MenuTracker has 76,405 records from 88 large out-of-home food chains at 4 different time points (ie, March, June, September, and December) in 2021. In constructing the database, we found that one-quarter (24.5%, 256/1043) of large chains, which are likely to be subject to the United Kingdom’s calorie menu labeling regulations, provided their nutritional information online in November 2021. Across these chains, 24.7% (16,391/66,295) of menu items exceeded the UK government’s recommendation of a maximum of 600 kcal for a *single meal*. Comparable figures were 46.4% (29,411/63,416) for saturated fat, 34.7% (21,964/63,388) for total fat, 17.6% (11,260/64,051) for carbohydrates, 17.8% (11,434/64,059) for sugar, and 35.2% (22,588/64,086) for salt. Furthermore, 0.7% to 7.1% of the menu items exceeded the maximum *daily* recommended intake for these nutrients.

**Conclusions:**

MenuTracker is a valuable resource that harnesses the power of data science techniques to use publicly available data online. Researchers, policy makers, and consumers can use MenuTracker to understand and assess foods available from out-of-home food outlets. The methods used in development are available online and can be used to establish similar databases elsewhere.

## Introduction

The consumption of food prepared out of the home is increasing worldwide. Eating outside of the home accounts for over half of food expenditures in the United States [1], 34% in Spain [2], and 27% in New Zealand [3]. In the United Kingdom, the percentage of total food expenditure outside the home was 28% in 2018/2019 [4]. In addition to dining out of the home, the rapid expansion of online delivery services also facilitates the consumption of food prepared outside the home. In an international study, 15% of respondents reported online delivery use [5]. The frequent consumption of food prepared out of the home is a public health concern as it is typically high in energy, salt, saturated fat, and sugar [6-11]. Frequent consumption of these foods has been associated with a higher BMI and an elevated risk of cardiovascular diseases [12,13].

The increasing frequency of eating out of the home makes food prepared by these chains an important avenue for improving population dietary quality. Internationally, decision makers are developing policies that promote healthier out-of-home options. The goal is to improve the out-of-home food environment and ensure that “the healthy choice is the easy choice” [14,15]. In the United Kingdom, for example, the government introduced the mandatory calorie menu labeling policy as part of a wider obesity strategy [16-18]. It requires large out-of-home food chains with 250 employees or more to add calorie labeling to menus for most of the food they sell starting from April 6, 2022 [17,19]. The effects of the policy may be not only to help consumers make informed choices but also to incentivize out-of-home chains to reformulate or provide healthier offerings [20].

Despite progress in policy, there are limited data on the nutrient composition of food prepared out of the home. As a recent World Health Organization report highlighted, a lack of quality data hinders the monitoring of the out-of-home food environment, creating barriers and challenges for policy development and evaluation [2]. With respect to the UK calorie labeling policy, a longitudinal nutritional database of food prepared out of the home is needed to investigate the direct effects of this policy on menus (eg, healthier menu options) and the overall effect on population dietary intake. In addition to aiding policy evaluations, nutrient composition data for out-of-home foods might also improve nutrient intake estimation in epidemiology studies by incorporating brand-specific information that is currently rarely included [21].

Many restaurants post nutritional information of their menu items online, and this information can be valuable for research. In the United States, a longitudinal restaurant nutritional database, MenuStat, was established in 2013 using information sourced from restaurant websites [22]. It has proven to be a valuable resource for researchers to advance the understanding of the restaurant food environment [23,24], assess changes in restaurant foods over time [25-28], and evaluate the potential impact of the calorie menu labeling policy in the United States [29]. Elsewhere, similar nutritional data for food and drinks prepared out of the home have been collected in New Zealand [30], Australia [31], and Canada [32]. However, to the best of our knowledge, these databases’ nutritional data were manually collected by researchers, and perhaps as a result, they have not been updated regularly, if at all. Manual collection of restaurant nutritional data by hand requires extensive human resources and is prone to error.

Web scraping, or automated data extraction from websites, provides an efficient, reliable, and flexible alternative to hand transcribing website data [33,34]. In the United Kingdom, web scraping has been used to establish a longitudinal nutritional database—foodDB—of packaged foods sold in large supermarkets [33]. Yet nutritional data are still limited for food prepared out of the home, largely due to the heterogeneity of how and what nutritional information is presented on out-of-home chains’ websites.

This study presents MenuTracker, the first longitudinal nutritional database, updated quarterly, of food prepared by large out-of-home food chains in the United Kingdom. In the future, we will use this database to describe and characterize changes in the nutrient content of out-of-home foods over time, to evaluate the effect of the calorie menu labeling policy, and potentially to improve nutrient intake estimation in nutritional epidemiology studies. In this paper, we aim to describe MenuTracker and its data collection methods, identify gaps in the presentation of nutritional information online, and demonstrate an example application of MenuTracker in food and nutrition research.

## Methods

### Overview

We have collated data on the nutritional composition of menu items sold by large UK food businesses (likely to be subject to the calorie labeling policy) from their websites quarterly since March 2021. We automated this data collection using web scraping techniques and PDF extraction tools. In the example application of this data, we examined the proportion of menu items exceeding recommended energy and nutrient intake values for the UK population.

### Out-of-Home Food Chain Inclusion Criteria

**Figure 1:**
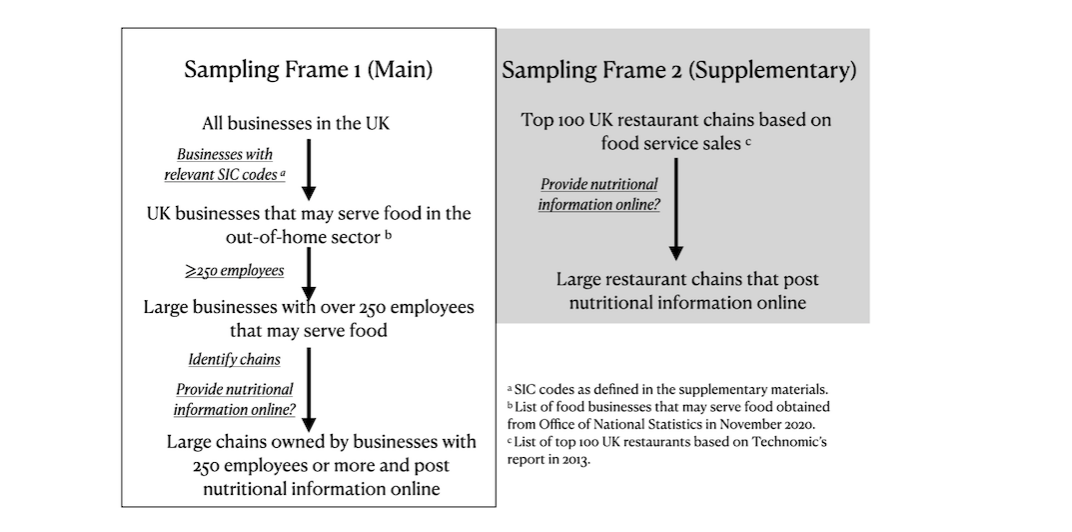
Out-of-home food chains inclusion criteria. SIC: Standard Industrial Classification.

Out-of-home food chains were defined as any chain where food or drink is prepared for immediate consumption by the person who buys it [18]. Figure 1 shows inclusion criteria for out-of-home food chains in MenuTracker. Essentially, two sampling frames were used for MenuTracker. Sampling frame one—the primary sampling frame—was a list of businesses that were potentially relevant (ie, may serve food) to the UK calorie menu labeling policy. In this study, we use the term business to refer to the parent company and chain to refer to the brands belonging to the businesses. We obtained the list from the Office for National Statistics (ONS) in October 2020. This list contained all businesses with Standard Industrial Classification (SIC) codes that indicated they might serve food (eg, “SIC 47.11: Retail sale in non-specialized stores with food, beverages or tobacco predominating”) and their employee numbers. A full list of included SIC codes included can be found in Multimedia Appendix 1. We then filtered to businesses with 250 employees or more and reviewed them to determine which of those provided nutritional information online. If there were multiple chains under one business, each chain was reviewed to determine the availability of online nutritional information. For example, under the business Mitchells & Butlers, there were more than 10 different chains, including Sizzling Pubs, Vintage Inns, Harvester, Ember Inns, and Toby Carvery. Each chain was reviewed and included if the chain provided nutritional information online. Sampling frame two—the supplementary sampling frame—contained the top 100 UK restaurants based on sales volume. Sales data were provided by Technomic, a market research company specializing in the food service industry, in 2013 [35]. This list of the top 100 food businesses supplemented our primary sampling frame to capture all large food businesses in the United Kingdom that may be eligible for calorie labeling. Each of these listed businesses were reviewed to determine whether they provided nutritional information online and would thus be included. Both lists are reviewed annually to check for changes in chains that provide nutritional information online.

### Menu and Menu Item Inclusion Criteria

All out-of-home menu items with online nutritional information were included in the data collection. In this paper, we used “nutritional information” to refer to “energy and nutritional information.” Menu item data were collected as they appeared on websites. We collected the out-of-home food chain name, menu item name, menu section, item description, serving size, and nutritional information. Additionally, ingredient statements, allergens, and dietary information (eg, vegetarian) were extracted if available on the same page or in the same PDF document.

When menus differed between locations (eg, Weatherspoon had different food menus at different locations), the first listed location in London was selected to represent the out-of-home chain. If the chain did not have a presence in London, a random location was selected. The same location for the chain was used in different data collection waves. When an out-of-home chain had different menus (eg, “Core” or “Delivery”), the main menu (eg, “Core Menu” or “Main Menu”) was used. The children’s menu and relevant promotional menus were also included in addition to the main menu, where available. If the nutritional document was last updated more than 3 years ago, it was deemed invalid. Only 1 restaurant was excluded due to this criterion.

Menu items of different sizes and beverages with different customization options were also included. For example, beverages with multiple choices of milk (eg, oat milk, soy milk, or whole milk) were entered as individual records, as well as pizza of different sizes (eg, individual, medium, large, or XXL). However, highly customizable menu items such as *building your own burritos* can lead to a large number of possible combinations. We collected the default customizations for these. If there were no default customizations, meal components for each menu item were collected and assigned an item ID for future linkage.

### Data Collection

**Figure 2:**
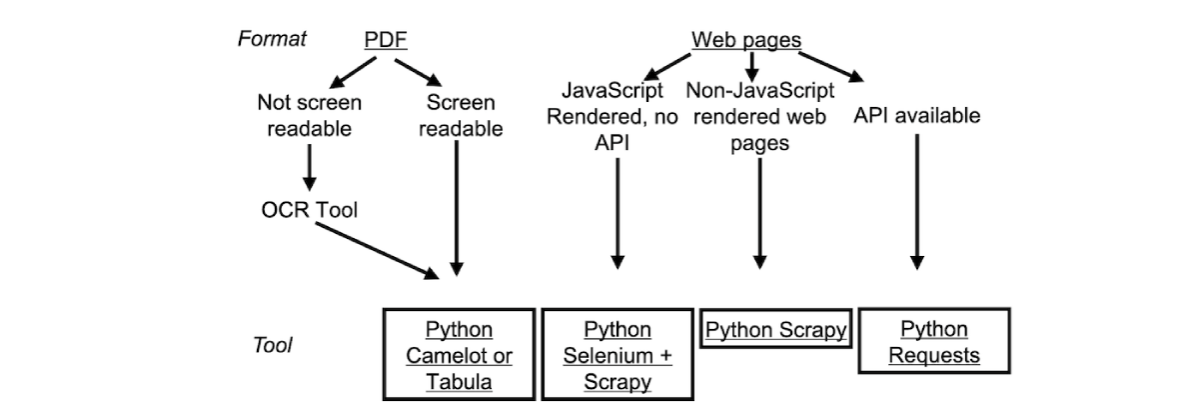
Restaurant Nutritional information formats and data collection tools. API: application programming interface; OCR: optical character recognition.

Before MenuTracker data collection, we collected four waves of data in a pilot study, which has been described in detail elsewhere [36,37]. Using the sampling frames described above, we used automated Python scripts to collect data for MenuTracker proper beginning March 2021. The codebase was developed from October 2020 to February 2021. Included chains presented their nutritional information directly on web pages or in separate downloadable PDF files. Despite the variations in how this information could be shown on web pages (eg, some were presented on individual item pages, while others were presented as nutrition tables separate from the item page), the web scraping fundamentals were the same. Hence, we can describe the web scraping method for all “web pages,” irrespective of how the nutritional information was presented (Figure 2).

For nutritional information presented in non–screen-readable PDF format, we first used the “Scan & OCR” tool in Adobe Acrobat to convert the PDF to be screen readable. We then used the Python packages Tabula or Camelot to extract data tables from PDFs. Both packages are designed to enable table extraction from PDFs, with Camelot allowing for more user customization and Tabula providing a more stable user interface to select table boundaries. The choice of package depended on the quality of output from these packages. In both packages, two table parsing methods, “stream” and “lattice,” were available. The “stream” parsing method estimates the number of columns based on row ranges and table areas. It performed better for PDF tables without clear boundaries and lines. The “lattice” parsing method defines tables based on table lines. It performed better for PDF tables with clear line segments. For each chain, we randomly selected one menu item in our data extract and compared to data on the website to ensure accuracy. We also checked all outliers for energy and nutrient values (eg, top/bottom 5%) in extracted data against websites.

Different data scraping methods were used to harvest nutritional information from web pages. For simple, non–JavaScript-rendered web pages, we used the Scrapy framework in Python to extract data. Scrapy is a powerful and highly customizable web scraping framework. However, the Scrapy framework alone cannot gather data from websites rendered by JavaScript. We used Selenium WebDriver within the Scrapy framework in these instances. There were a few websites where nutritional data were loaded through application programming interface (API) requests. An API is built for information retrieval, enabling data transmission between software. For example, when a web page is being loaded, the web server requests data from the company’s database/server through the API. For websites where nutritional information was loaded through an API (identified through the inspection of developer tools in Chrome), we used the Python Request library to pull data directly.

We abided by the UK ONS’s safe web scraping policy to minimize the burden of our extraction on web site owners [38]. Additionally, we worked within what is allowed from a copyright perspective. The UK government’s guidance on copyright outlines several exceptions, including limited use of copyright works for noncommercial research studies [39]. Scripts were checked quarterly and updated to accommodate any changes in web site structures that may have occurred since the previous scrape. The most up-to-date scripts are available publicly on GitHub [40].

### Data Cleaning and Standardization

To address inconsistency in the portion size information for pizzas prepared by large pizza chains such as Pizza Hut and Papa John’s, we calculated the energy and nutrient values for 3 slices of pizza if an item was described as “large,” “family,” “for sharing,” or “medium,” and the whole pizza if an item was described as “small” or “individual.” This was in accordance with the way Domino’s—the leading pizza chain in the United Kingdom—presented the nutritional information on its pizzas. We also saved the original energy and nutrient values for pizza items.

The field names were also standardized for each out-of-home food chain. For example, “sugar,” “sugar content,” and “sugars” were all standardized as “sugar.” Operators in the nutrition values were also removed before converting to numeric values. As an example, “<0.05” was replaced with “0.05” for conservative estimates. Nutrition values with “-” or blanks were set to missing. All verbatim texts (including operators) were stored in each restaurant’s data collection folder.

After standardization and data cleaning, we compiled all data into one master file for each quarterly data collection.

### Energy and Daily Nutrient Intake Values

For our example application of MenuTracker data, we estimated the proportion of menu items exceeding the United Kingdom’s per meal recommendations and daily reference intakes in 2021. The daily reference intakes for an adult are 2000 kcal for energy, less than 70 g for total fat, less than 20 g for saturated fat, 260 g for carbohydrates, 90 g for total sugars, and less than 6 g for salt [41]. The reference intake values for energy and nutrients are calculated based on an average female with an average amount of physical exercise. The UK government recommends adults also consume no more than 600 kcal for lunch or dinner [42]. Although there are no specific *per meal* recommendations for other nutrients, it has been suggested that any meal components should not exceed 30% of daily reference intake, in line with the UK government guidelines [43]. As such, we set the *per meal* recommendations for total fat, saturated fat, carbohydrates, sugars, and salt at 30% of the daily reference intakes, proportional to the energy recommendations. We used all MenuTracker records collected in 2021 for this analysis.

## Results

### Availability of Calorie Information Among Large Out-of-Home Food Chains

A total of 1043 businesses with 250 employees or more were identified in October 2020. This represents a likely overestimate of the number of businesses potentially eligible for the UK calorie labeling policy (eg, not all “historical sites and buildings and similar visitor attractions” in SIC 91.03 served food). Among these 1043 businesses, 256 (24.5%) presented nutritional information for their menu items (food prepared out of the home) available online in November 2021. Companies operating as franchisees of other businesses (n=196) typically serve the same menu as provided by the franchisor. As such, data on franchisees were not collected unless the main chain had not been captured (n=3; eg, Taco Bell UK).

In total, 82 unique chains provided nutritional information online using the main sampling frame in March 2021. The supplementary sampling frame added 3 additional food chain brands (ie, Papa John’s, PAUL, and Ben & Jerry’s). This gave a total of 85 unique chains.

### Data Collection Automation

In our pilot study, it was estimated to have taken one researcher 36 working days to collect and transcribe data from 42 out-of-home food chains in 2018 [44]. Using automated programs written in Python, we were able to collect data from around 85 food chains in about 10 working days. This is an 85% reduction in hours, totaling approximately 500 hours, compared to the manual transcription in 2018.

### Descriptive Statistics

As shown in Table 1, a total of 85, 83, 79, and 81 out-of-home food chain brands were included in MenuTracker across March, June, September, and December 2021, respectively. A list of all included chains as of March 2021 can be found in Multimedia Appendix 2. The number of included food chains varied as some out-of-home food chains stopped providing nutritional information online while others started during 2021. Some chains did not provide nutritional information for every menu item listed. Among menu items with calorie information (86.1-87.6% of all items identified), information on fat, saturated fat, carbohydrates, sugars and salt were available for the majority (94.6-97.5%). However, only 36.7%-42.4% of items with calorie information had associated serving size information and around half of them provided fibre information.

**Table 1:** MenuTracker 2021 data summary statistics.

			March 2021		June 2021		September 2021		December 2021
Out-of-home chains, n			85		83		79		81
Menu items, n			18,005		19,310		19,392		19,698
**Item-level availability**									
	Energy, n	15,766		16,678		16,882		16,969	
	Fat, n (%)^a^	15,244 (96.7)		15,785 (94.6)		16,069 (95.2)		16,290 (96.0)	
	Saturated fat, n (%)^a^	15,261 (96.8)		15,774 (94.6)		16,028 (94.9)		16,353 (96.4)	
	Carbohydrates, n (%)^a^	15,183 (96.3)		16,021 (96.1)		16,308 (96.6)		16,539 (97.5)	
	Sugars, n (%)^a^	15,233 (96.6)		16,028 (96.1)		16,279 (96.4)		16,519 (97.3)	
	Protein, n (%)^a^	15,160 (96.2)		15,777 (94.6)		16,078 (95.2)		16,194 (95.4)	
	Salt, n (%)^a^	15,179 (96.3)		16,009 (96.0)		16,357 (96.9)		16,541 (97.5)	
	Fiber, n (%)^a^	8167 (51.8)		8367 (50.2)		8750 (51.8)		8229 (48.5)	
	Serving weight, n (%)^a,b^	6348 (40.3)		6721 (40.3)		7153 (42.4)		6235 (36.7)	

^a^Percentage per items that provided calorie information.

^b^Items that provided serving size information directly or if the information can be calculated through nutrient per serving and nutrient density.

### Proportion of Menu Items Exceeding Per Meal and Daily Reference Intake

**Figure 3:**
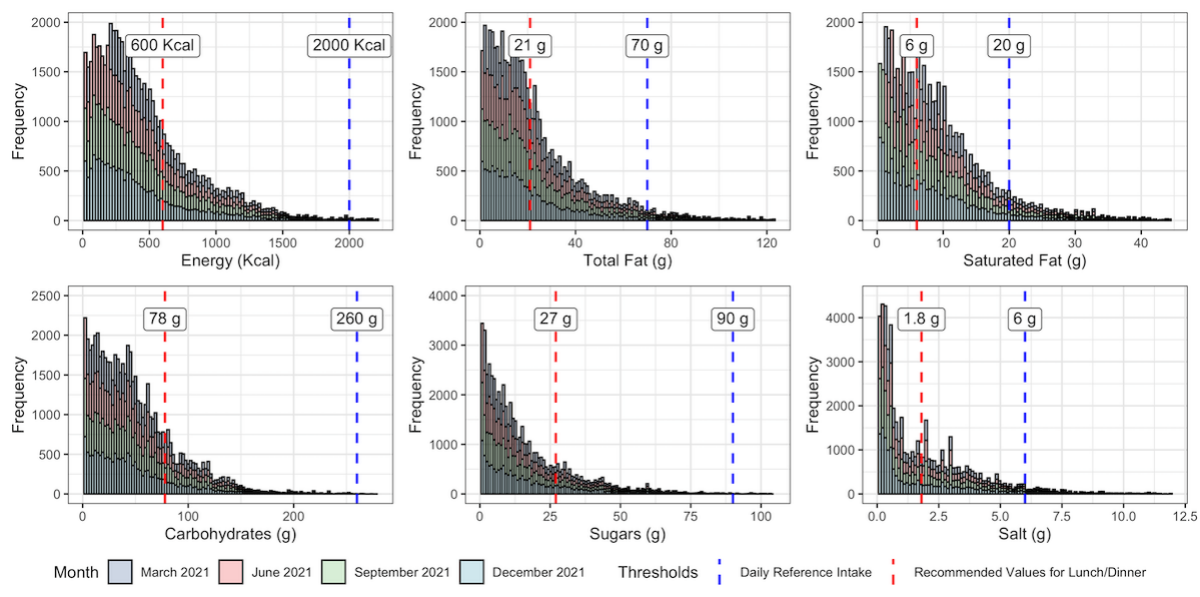
Menu item energy and nutrient distributions.

As shown in Figure 3, the largest proportion of any nutrient exceeding allowances was saturated fat, where 46.4% (29,411/63,416) of menu items exceeded per meal recommendations and 7.1% (4523/63,416) exceeded the daily reference intake. Proportions of menu items exceeding the per meal recommendation for salt, total fat, energy, sugar, carbohydrates were 35.2% (22,588/64,086), 34.7% (21,964/63,388), 24.7% (16,391/66,295), 17.8% (11,434/64,059), and 17.6% (11,260/64,051), respectively. Comparable figures for proportions of items exceeding daily reference intakes were 0.7% (497/66,295) for energy, 3.6% (2258/63,388) for total fat, 0.1% (75/64,051) for carbohydrates, 0.4% (245/64,059) for sugar, and 4.2% (2722/64,086) for salt. Detailed proportions by different data collection waves can be found in Multimedia Appendix 3.

## Discussion

### Summary of Findings

In this study, we described MenuTracker, the first longitudinal nutritional database of food prepared by out-of-home food chains in the United Kingdom who provided this information online. As of December 2021, MenuTracker includes 76,405 menu item records from over 80 large out-of-home food chains, collected across 4 time points. The database is semiautomated and time stamped. In constructing the database, we found that less than one-quarter of businesses that might be subject to a calorie menu labeling policy in the United Kingdom had presented menu item energy information on their websites as of November 2021. Across chains that provided nutritional information online, a large proportion of menu items did not have associated serving weight or nutrient density information. Using MenuTracker data, we found that more than one-third of items available in the database were high in saturated fat, total fat, or salt, and one-fourth were high in energy in comparison to recommendations for a main meal.

### Interpretation of Findings

#### Uniqueness of the Database

MenuTracker data represents a valuable resource for dietary public health and nutrition research, as well as policy making in this space. As it is regularly updated and data are time stamped, it allows researchers and policy makers to track nutritional composition changes in the UK out-of-home food environment over time. The nutritional data contained are harvested directly from the official websites of food chains in a systematic fashion, ensuring accuracy. Among other potential future applications, MenuTracker will enable the evaluation of calorie menu labeling, assessments of the out-of-home food environment, and refinement of nutrient estimation in nutritional epidemiologic studies. In the UK policy context, few obesity policies have been proposed with an evaluation plan and MenuTracker data may facilitate policy evaluations [45].

Manual collection of nutritional composition data is labor-intensive. In 2018 and 2019, researchers in our group hand transcribed MenuTracker data annually. The automation codebase reduced 85% of manual work hours, allowing us to continue collecting MenuTracker data every quarter. We currently have resources to continue to collect MenuTracker data at least until spring 2023.

#### Out-of-Home Food Chains Nutrient Reporting in the United Kingdom

In the United Kingdom, starting from April 6, 2022, the mandatory calorie labeling policy is now in effect [19]. In this study, we found less than one-quarter of potentially eligible businesses posted calorie information on their websites in November 2021. This is broadly consistent with a previous UK study in 2018 where only 17% of large chains were found to provide calorie labeling in store [46]. However, our calculated percentage could be an underestimation, as some of these out-of-home businesses could be exempt from the calorie menu labeling policy (eg, seasonal items only), or they may not serve food at all. Nonetheless, our results highlight a gap in nutrient reporting for out-of-home chains before the regulations came into effect, which may indicate the industry’s reluctance to present this information voluntarily.

Notably, for out-of-home food chains that post nutritional composition online, nutrient density (eg, kcal per 100 g) information (or serving weight, which would permit its calculation) was missing for around 60% of items. As most voluntary reduction programs (eg, for salt and sugar) set targets based on nutrient density, this may prohibit monitoring and evaluation of these initiatives [47,48]. Moreover, this information is critical for identifying menu item reformulations—a key potential impact of menu labeling regulation—as any overall change in nutrient content may be caused by reformulation or change in serving size. Without serving size indicators, these possibilities cannot be distinguished. Mandating the declaration of serving sizes (alongside calorie information) could enable more comprehensive evaluations of interventions targeting the out-of-home food retail sector.

#### Example Use Cases of MenuTracker

In this study, we demonstrated an example application of MenuTracker data. We used MenuTracker data to estimate the proportions of menu items excessively high in energy and nutrient content. The proportion of menu items exceeding the per meal energy recommendation was broadly similar to that previously reported in the United Kingdom [6,7]. Our results also draw attention to other nutrients high in out-of-home food, such as saturated fat and salt. Our data reaffirms that in 2021 food prepared by large out-of-home chains were high in energy and nutrients such as sugars (for which intake should be limited).

In a recent paper, we demonstrated the feasibility of using MenuTracker to monitor changes in the nutritional composition of food prepared out of the home over time [36]. Elsewhere, we used US MenuStat (equivalent to MenuTracker) data to draw international comparisons in the nutritional composition of food away from home [49]. These applications of MenuTracker demonstrate its power as a research tool.

### Limitations and Future Directions

While most of the MenuTracker data collection has been automated, manual review and modification of code are still needed at each wave of data collection. This need arises due to two main issues: challenges of data extraction from PDF documents and changing web site structures. PDF conversion tools are imperfect and unable to correctly identify table boundaries at times, which necessitates the manual checking of results. Web site structures and design are also subject to change, which requires the updating of paths for certain elements or the rewriting of scripts. Currently, we monitor the changes in web site structures each quarter to ensure the codebase works properly for each data wave. However, as more chains start providing nutritional information on their web pages, along with advances in PDF conversion tools, full automation might be achievable in the future. Alternatively, as the calorie labeling regulations extend to third-party delivery platforms such as Just Eat and Deliveroo, it would be less resource-intensive to collect calorie information from these delivery platforms, compared to 80 individual websites. In the future, we may transition to obtaining calorie information in this way. However, at this time, information on other nutrients remains unavailable on these platforms, meaning that such a transition would lead to loss of the breadth of information.

MenuTracker itself is not without limitations. MenuTracker focuses on large out-of-home food chains and does not include energy and nutritional information from smaller chains or independent businesses. However, the UK Government estimates that these large chains make up 50% of all out-of-home food and drink sales [18]. Additionally, MenuTracker focuses on online menus from chains’ official web sites, which may differ from the physical menus in-store or on delivery platforms. This could be important, as throughout the COVID-19 pandemic, use of online food delivery services has increased worldwide [50,51]. Future research is needed to understand potential differences between online menus from chains’ official web sites, in-store menus, and menus on delivery service web sites. Moreover, chains and menu items with online nutritional information may also have different characteristics compared with those without. Future research could also explore what types of chains and menu items are more likely to have the full energy and nutritional information.

Another limitation relates to our sampling frame. The list of food businesses we obtained was for October 2020, and businesses are likely to have both been added to and dropped from the list since then. To mitigate this concern, we will review this list annually. However, there remains the possibility that new large businesses have not been subsequently included in MenuTracker. Moreover, the fact that only one-quarter of businesses potentially eligible for the calorie labeling policy provided nutritional information online undermines the market coverage of MenuTracker. As the calorie labeling policy is now in effect, MenuTracker will be expanded to include new out-of-home food chains that start providing relevant information—although this may well be limited to energy information only. Moreover, MenuTracker relies on self-reporting of nutritional information by chains, which may not be entirely accurate. However, we believe that these large chains have the incentive to provide accurate nutritional information. Lastly, the nutritional information presented on chain web sites may be outdated. It was difficult for us to determine when and how nutritional information was obtained for each chain if no time stamp was provided.

In addition to the inclusion of new businesses and potentially obtaining data from delivery platforms, MenuTracker will benefit in the future from the development of machine learning models for the categorization of menu items and automated linkage. This will allow category-specific (eg, food and drinks) tracking of energy and nutrient content over time while saving hundreds of hours of manual labeling for each data wave. We piloted a record linkage process in our recent paper, which was used to track energy and nutrient changes for the same set of menu items over time [36]. We plan to refine this technique and implement record linkage in existing and future MenuTracker data.

### Conclusions

Using data science techniques, we established MenuTracker, a valuable database for researchers and policy makers to understand and assess foods available from large chains in the United Kingdom who provide this information online. In constructing the database, we found less than one-quarter of chains potentially eligible for the calorie labeling policy provided nutritional information online, and serving size information was missing for a large proportion of menu items in 2021. This may present challenges for monitoring the out-of-home food environment. This study also adds to the growing body of evidence suggesting that foods prepared out of the home are high in saturated fat, total fat, and salt in the United Kingdom. The methods used in development are available online and can be used to establish similar databases elsewhere.

## Appendix

Multimedia Appendix 1 Standard Industrial Classification codes used for selecting businesses that may serve food.

Multimedia Appendix 2 Chains included in MenuTracker, March 2021.

Multimedia Appendix 3 Proportion of menu items exceeding per meal and daily reference intake by data collection wave.

## Abbreviations

API: application programming interface
ONS: Office for National Statistics
SIC: Standard Industrial Classification

## Data Availability

The codebase is publicly available on GitHub [40]. All analysis codes are also available upon request. The anonymized data set used in the analyses are available on request. Use of our data is only permitted for noncommercial purposes.
