# Supplementary material for: Monitoring the Nutrient Composition of Food Prepared Out-of-Home in the United Kingdom: Database Development and Case Study
Source: JMIR Public Health Surveill. 2022 Sep 8;8(9):e39033. doi: 10.2196/39033 (PMC9501650; doi:10.2196/39033)
Supplement: Multimedia Appendix 1 [file publichealth_v8i9e39033_app1.docx]

## Standard Industrial Classification of Economic Activities (SIC) codes used for selecting businesses that may serve food

· SIC 2007 section I (accommodation and food service activities)

· Within section G (Wholesale and retail trade; repair of motor vehicles and motorcycles):

o SIC 47.11- Retail sale in non-specialised stores with food, beverages or tobacco predominating.

o SIC 47.24- Retail sale of bread, cakes, flour confectionery and sugar confectionery in specialised stores.

o SIC 47.29 Other retail sale of food in specialised stores.

· Within section R (Arts, entertainment and recreation):

o SIC 91.03-Operation of historical sites and buildings and similar visitor attractions

o SIC 91.04-Botanical and zoological gardens and nature reserve

o SIC 93.11-Operation of sports facilities

o SIC 93.12-Activities of sports clubs

o SIC 93.13-Fitness facilities

o SIC 93.21-Activities of amusement parks and theme parks

· Within section J (Information and communication):

o SIC 59.14-Motion picture projection activities

## 
