# Supplementary material for: Monitoring the Nutrient Composition of Food Prepared Out-of-Home in the United Kingdom: Database Development and Case Study
Source: JMIR Public Health Surveill. 2022 Sep 8;8(9):e39033. doi: 10.2196/39033 (PMC9501650; doi:10.2196/39033)
Supplement: Multimedia Appendix 2 [file publichealth_v8i9e39033_app2.docx]

Chains included in MenuTracker, March 2021:

| All Bar One | Farmhouse Inns | Sizzling Pubs | Zizzi |
| --- | --- | --- | --- |
| Asda Cafe | FIVE GUYS | Soho Coffee |  |
| Ask | Flaming Grill Pub Co. | Starbucks |  |
| Barburrito | GBK | Stonehouse Pizza & Carvery | |
| Beefeater Grill | Greene King | Subway |  |
| Bella Italia | Greggs | Table Table |  |
| Ben & Jerry's | Harvester | Taco Bell |  |
| Benugo Cafe | Itsu | Tank and Paddle | |
| Bill's | JOE & THE JUICE | Tesco Cafe |  |
| Birds Bakery | KFC | The Cornish Bakery | |
| Boost Juice Bars | Krispy Kreme | The Real Greek | |
| BOSWELL | Leon | Thomas the Baker | |
| Brewers Fayre | Loch Fyne | Tim Hortons |  |
| Brewhouse and Kitchen | Marston's | Toby Carvery | |
| Burger King | McDonalds UK | Top Golf |  |
| Caf‚àö¬© Rouge | Morrisons Cafe | Tortilla |  |
| Caffe Nero | Nandos | Tossed |  |
| Chef and Brewer | ODEON | Town, Pub & Kitchen | |
| Chicken Cottage | Papa John's | Vintage Inns | |
| Cineworld | PAUL | VUE ENTERTAINMENT | |
| Coco Di Mama | Pho | Wagamama | |
| Coffee #1 | Pieminister | Walkabout |  |
| Common Room | Pizza Hut | Wasabi |  |
| Cookhouse & Pub | PizzaExpress | Waterfield's | |
| Costa Coffee | Pret A Manger | Wetherspoon | |
| Crussh | Pure. | Wimpy |  |
| Domino's Pizza | Revolution Vodka Bars | Yate's |  |
| Ember Inns | Sainsbury's | YO! Sushi |  |
