# Supplementary material for: Monitoring the Nutrient Composition of Food Prepared Out-of-Home in the United Kingdom: Database Development and Case Study
Source: JMIR Public Health Surveill. 2022 Sep 8;8(9):e39033. doi: 10.2196/39033 (PMC9501650; doi:10.2196/39033)
Supplement: Multimedia Appendix 3 [file publichealth_v8i9e39033_app3.docx]

## Proportion of menu items exceeding per meal and daily reference intake by data collection wave

| Month | March | June | September | December |
| --- | --- | --- | --- | --- |
| Exceeding *Per Meal* Recommendation (%) | | | | |
| Calories | 25.0% | 24.4% | 27.1% | 22.4% |
| Fat | 35.1% | 34.3% | 36.8% | 32.4% |
| Saturated Fat | 46.0% | 45.6% | 47.5% | 46.4% |
| Carbohydrates | 18.0% | 17.0% | 19.5% | 15.9% |
| Sugar | 17.0% | 17.6% | 17.4% | 19.3% |
| Protein | 38.2% | 38.2% | 41.1% | 35.4% |
| Salt | 35.8% | 34.1% | 37.8% | 33.2% |
| Exceeding *Daily* Nutrient Intake (%) | | | | |
| Calories | 0.6% | 0.7% | 0.8% | 0.8% |
| Fat | 3.6% | 3.4% | 3.9% | 3.4% |
| Saturated Fat | 6.8% | 7.2% | 7.8% | 6.8% |
| Carbohydrates | 0.1% | 0.1% | 0.1% | 0.1% |
| Sugar | 0.4% | 0.4% | 0.4% | 0.4% |
| Protein | 7.2% | 7.2% | 7.3% | 6.3% |
| Salt | 4.3% | 3.9% | 4.5% | 4.2% |
